# Regulation of Primary Metabolic Pathways in Oyster Mushroom Mycelia Induced by Blue Light Stimulation: Accumulation of Shikimic Acid

**DOI:** 10.1038/srep08630

**Published:** 2015-02-27

**Authors:** Masanobu Kojima, Ninako Kimura, Ryuhei Miura

**Affiliations:** 1 Department of Bioscience and Biotechnology, Shinshu University, Minamiminowa, Kamiina, Nagano, Japan

## Abstract

Shikimic acid is a key intermediate in the aromatic amino acid pathway as well as an important starting material for the synthesis of *Tamiflu*, a potent and selective inhibitor of the neuraminidase enzyme of influenza viruses A and B. Here we report that in oyster mushroom (*Pleurotus ostreatus*) mycelia cultivated in the dark, stimulation with blue light-emitting diodes induces the accumulation of shikimic acid. An integrated analysis of primary metabolites, gene expression and protein expression suggests that the accumulation of shikimic acid caused by blue light stimulation is due to an increase in 3-deoxy-D-arabinoheptulosonate 7-phosphate synthase (DAHPS, EC2.5.1.54), the rate-determining enzyme in the shikimic acid pathway, as well as phosphofructokinase (PFK, EC2.7.1.11) and glucose-6-phosphate dehydrogenase (G6PD, EC1.1.1.49), the rate-determining enzymes in the glycolysis and pentose phosphate pathways, respectively. This stimulation results in increased levels of phosphoenolpyruvic acid (PEP) and erythrose-4-phosphate (E4P), the starting materials of shikimic acid biosynthesis.

Light is an important environmental cue for regulating growth and morphogenesis of organisms, as well as the production of secondary metabolites. In plants, it is known that phytochromes, which are red and far-red light photoreceptors, together with phototropins and cryptochromes, which are blue light photoreceptors, participate in the regulation of growth and morphogenesis^1^,^2^. Recent studies have also identified red and blue light photoreceptors in fungi^3^,^4^,^5^,^6^,^7^, as well as photoresponse phenomena related to fruit body formation^7^,^8^, pigmentation^9^,^10^,^11^, sporulation^9^,^10^, phototoropism^10^,^12^, circadian cycle^13^ and biosynthesis of secondary metabolites^9^,^14^. To our knowledge, however, there have been no studies on the regulation of primary metabolites caused by light stimulation in fungi or other organisms.

Our research group has reported that blue light stimulation suppresses the growth of oyster mushroom mycelia, and causes the up- and down-regulation of twenty-eight photoresponsive genes^15^,^16^. In this study, we investigated how blue light stimulation affects the concentration of primary metabolites in oyster mushroom mycelia. We found that shikimic acid, a key intermediate in the aromatic amino acid pathway^17^ and an essential starting material in the synthesis of *Tamiflu*, an influenza neuraminidase inhibitor^18^,^19^,^20^, accumulates more than two-hundred fold when compared to oyster mushroom mycelia kept in the dark. We measured the accumulation of shikimic acid based on stimulation time and light intensity, and determined the correlations between the expression levels of three genes and the accumulation of their corresponding product metabolites. The three genes encoded the following enzymes: i) DAHPS, a rate-determining enzyme in the shikimic acid pathway^21^; ii) PFK, a rate-determining enzyme in the glycolysis pathway^22^ and providing PEP as the product, and iii) G6PD, a rate-determining enzyme in the pentose phosphate pathway and providing E4P as the product^23^. The levels of the proteins translated from the above genes were also determined. Our data demonstrate that shikimic acid accumulates in oyster mushroom mycelia stimulated with blue light, and that this accumulation is due to increases in the amounts of these three rate-determining enzymes. In addition, this approach has considerable potential for the production of useful primary and secondary metabolites using the aromatic amino acid pathway in filamentous fungi^24^.

## Results and Discussion

### Dynamics of primary metabolites in oyster mushroom mycelia stimulated by blue light

**Figure 1:**
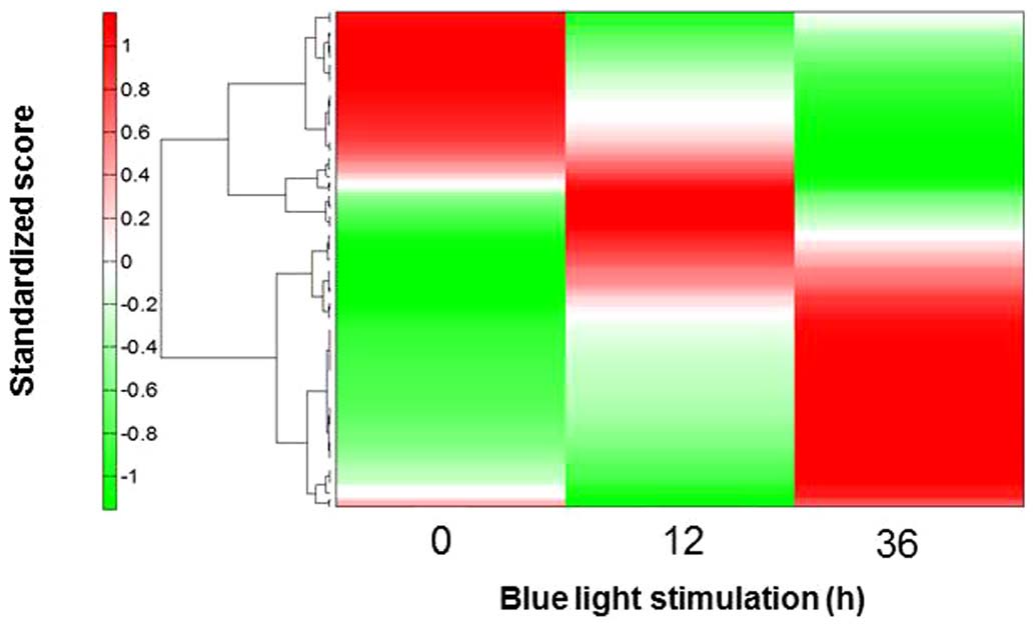
Dynamics of the primary metabolites in oyster mushroom mycelia during blue light stimulation: PFD, 150 μmol m^−2^ s^−1^; stimulation time, 0, 12 and 36 h. Plot of standardized score vs. blue light stimulation: n = 1.

Approximately 65-mm diameter oyster mushroom mycelial colonies on GPY agar media were cultured in the dark, then illuminated for 0.5 to 72 h with blue light (peak emission wavelength (PEW): 470 nm; spectrum radiation bandwidth (SRB): 30 nm; photon flux density (PFD): 150 μmol m^−2^ s^−1^) using a light-emitting diode (LED) lighting unit with an attached blue LED panel. Primary metabolites in irradiated colonies were analyzed by capillary electrophoresis time-of-flight mass spectrometry (CE-TOF-MS) and HPLC (Supplementary Fig. S1) as described in the Methods and their levels were compared with those in non-irradiated colonies. Hierarchical cluster analysis (HCA) of the time course of the primary metabolites using oyster mushroom mycelial colonies illuminated for 0, 12 and 36 h showed that major metabolites accumulated in the mycelia before illumination (0 h) and thus provided higher values than the standardized score (red bands in Fig. 1); interestingly, these metabolites tended to decrease with increasing light stimulation time. In contrast, the minor metabolites in the mycelia before illumination (green band in Fig. 1) tended to increase with light stimulation. A more detailed HCA is provided in Supplementary Table S1.

**Figure 2:**
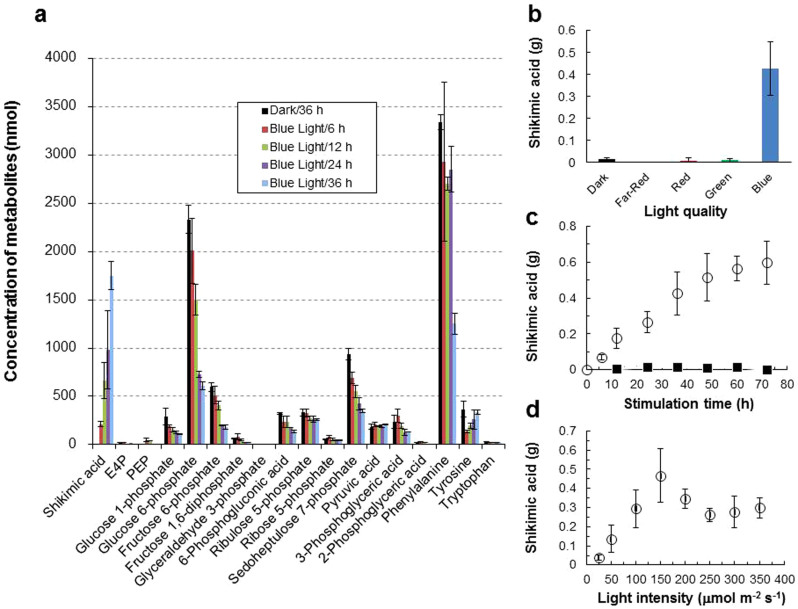
Effects of blue light stimulation on the biosynthesis of primary metabolites in oyster mushroom mycelia at 15°C. (a).Time course of the increase or decrease in the concentration (nmol) of metabolites related to shikimic acid biosynthesis in 1 g of the mycelia, determined by CE-TOF-MS. PFD, 150 μmol m^−2^ s^−1^; stimulation time, 0–36 h; n = 2; error bar = SE. (b). Effects of light quality on the accumulation of shikimic acid (g) in 1 kg of the mycelia, determined by HPLC. PFD, 150 μmol m^−2^ s^−1^; light stimulation time, 36 h; n = 3–8; error bar = SD. (c). Effects of blue-light stimulation time on accumulation of shikimic acid (g) in 1 kg of the mycelia, determined by HPLC. PFD, 150 μmol m^−2^ s^−1^; n = 5–8; error bar = SD; irradiated mycelia (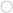), non-irradiated mycelia (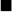). (d). Effects of blue light intensity on accumulation of shikimic acid (g) in 1 kg of the mycelia, determined by HPLC. Light stimulation time, 36 h; n = 8; error bar = SD.

**Figure 3:**
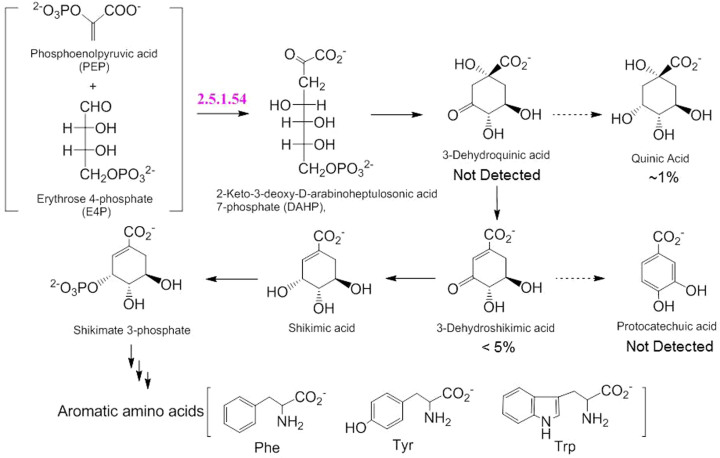
Shikimic acid pathway. Condensation of PEP and E4P, which are synthesized in the glycolysis and pentose phosphate pathways, respectively, is catalyzed by DAHPS (EC2.5.1.54), a rate-determining enzyme in the shikimic acid pathway.

Importantly, the primary metabolite exhibiting the largest increase with blue light stimulation was shikimic acid (Fig. 2a,c), a key intermediate in the aromatic amino acid pathway (Fig. 3). Furthermore, oyster mushroom mycelial colonies were illuminated with far red (PEW: 735 nm; SRB: 30 nm), red (PEW: 660 nm; SRB: 20 nm) and green (PEW: 525 nm; SRB: 30 nm) LEDs at a PFD of 150 μmol m^−2^ s^−1^, and the shikimic acid content in the colonies was measured using HPLC (Supplementary Fig. S1). The results indicated that blue light, but not far red, red or green light, stimulates the accumulation of shikimic acid (Fig. 2b). The increase in shikimic acid content caused by blue light stimulation at a PFD of 150 μmol m^−2^·s^−1^ with time was almost linear until 36 h, then slowed down (Fig. 2c). The optimal blue light PFD for stimulating the accumulation of shikimic acid was thus determined to be approximately 150 μmol m^−2^ s^−1^ for 36 h (Fig. 2d).

### Microarray and quantitative PCR analyses of gene expression induced by blue light stimulation

Using DNA sequences for 12,330 predicted transcript models obtained from the Joint Genome Institute (Pleurotus Ostreatus PC15 v2.0, http://genome.jgi-psf.org/PleosPC15_2/PleosPC15_2.home.html)^25^, 12,244 probes for a custom microarray were designed for 12,244 target transcripts (one probe per transcript). Comprehensive analysis of gene expression in the mycelial colonies after blue light stimulation at a PFD of 150 μmol m^−2^ s^−1^ was performed using the microarray. Eight clusters were categorized based on the expression patterns, as shown in Supplementary Fig. S2. Clusters I and V (total number of genes grouped (TNG): 110 and 119) increased significantly with stimulation time. Conversely, Clusters IV (189) and VI (556) decreased distinctly with increasing stimulation time. The changes in the expression levels for Clusters II (735), III (267), VII (534) and VIII (1165) were relatively small. In particular, the time course of the expression levels for Clusters I and V, as well as Clusters IV and VI, showed good correlations with the standardized scores for the primary metabolites, as seen in Fig. 1. Detailed HCA of the time course expression of genes annotated in oyster mushroom and related to aromatic amino acid biosynthesis, glycolysis/gluconeogenesis, and the pentose phosphate pathway was performed. The results, shown in Supplementary Table S2 and Supplementary Fig. S3, indicate that stimulation of the mushroom mycelia with blue light can induce the up- and down-regulation of the expression of many genes simultaneously.

**Figure 4:**
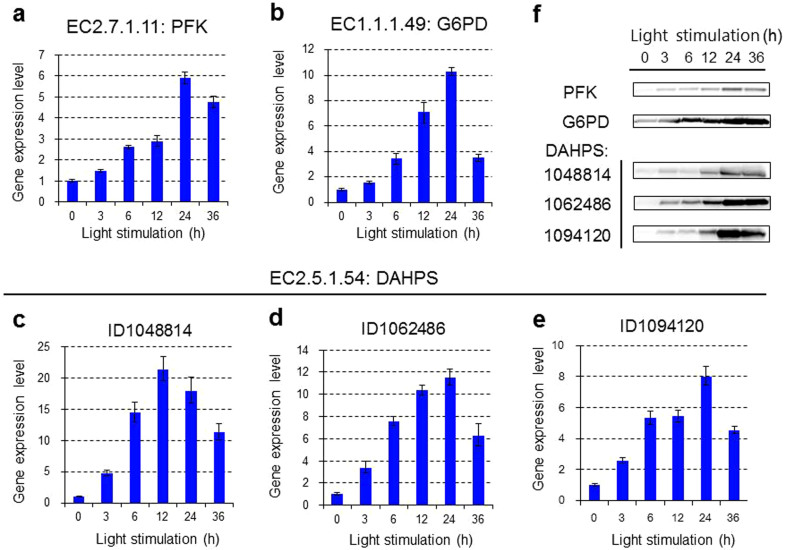
Real-time PCR analysis of the expression of genes encoding EC2.7.1.11 (PFK, a), EC1.1.1.49 (G6PD, b) and EC2.5.1.54 (DAHPS, c–e) and dynamics of these rate-determining enzymes (f) related to the biosynthesis of shikimic acid during blue light stimulation. Three isozymes were identified for DAHPS (IDs: 1048814, 1062486 and 1094120); (f). Time course of the expression levels of the rate-determining enzymes detected by antibody response.

As shown in Fig. 3, shikimic acid is biosynthesized by the shikimic acid pathway through a condensation reaction between PEP and E4P, catalyzed by DAHPS. The starting materials are biosynthesized by the glycolysis and pentose phosphate pathways, respectively (Supplementary Figs. S4 and S5). Analysis of the oyster mushroom DNA sequence identified homologous proteins for PFK and G6PD: protein IDs 174437 and 1088946, respectively. In addition, three isozyme proteins (IDs: 1048814, 1062486 and 1094120) were identified as DAHPS. The expression levels of the genes encoding the above rate-determining enzymes after blue light stimulation were precisely determined by quantitative PCR using forward and reverse primers designed with Primer3Plus (Supplementary Table S3)^26^. As shown in Fig. 4a–e, blue light stimulation induced increases in the expression levels of the genes encoding PFK, G6PD and DAHPS.

### Protein expression induced by blue light stimulation

We further confirmed the protein levels of PFK, G6PD and DAHPS. The amino acid sequence of each protein was predicted from its gene sequence, then polyclonal primary antibodies against the target proteins were prepared using synthesized peptides (Supplementary Table S4 and Methods). The expression levels of these three proteins induced by blue light stimulation were semi-quantitatively analyzed using Western blotting. As shown in Fig. 4f and Supplementary Fig. S6, the expression levels of the three rate-determining enzymes clearly increased, in accordance with the gene expression levels.

### Blue light signaling pathway

WC-1 and cryptochrome have been found as blue light photoreceptors in *Neurospora crassa*, a member of the class Ascomycetes^4^. Oyster mushroom belongs to the class Basidiomycetes. The WC-1 homologue has been identified in several Basidiomycetes as a blue light photoreceptor: for example, Dst1 in *Coprinus cinereus* and Bwc1 in *Cryptococcus neoformans*^4^. However, the gene encoding the WC-1 homologue in oyster mushroom has not been found or annotated. Although blue light photoreceptors in oyster mushroom remain to be identified, blue light clearly must interact with photoreceptors and act as a signal to mushroom DNA. Following the transcription of DNA into messenger RNAs (mRNAs) in response to the stimulation, the mRNAs are then translated into polypeptide chains, which ultimately fold to provide functional proteins. Consequently, many primary metabolites in the mushroom mycelia must have been up- or down-regulated in tandem with the corresponding changes in protein expression levels. Although it is currently unclear why mushroom mycelia accumulate shikimic acid upon blue light stimulation, further studies to clarify the detailed mechanism are in progress in our laboratory.

### Replacing shikimic acid production from plant sources and recombinant microbes with the photoregulation of primary metabolite pathways in mushroom mycelia

To date, shikimic acid has been mainly isolated from *Illicium* *verum*^17^. However, due to the clinical efficacy of *Tamiflu* as a neuraminidase inhibitor^18^,^19^,^20^, there is strong demand for chiral starting materials for the synthesis and development of *Tamiflu* and derivative drugs. Shikimic acid production methods based on recombinant microbial biocatalysis^27^, oxidative fermentation^28^, and organic synthesis^29^,^30^ have been developed to provide a sustainable and economically viable supply; however, obtaining highly pure shikimic acid at low cost remains challenging. Recently, synthetic routes for *Tamiflu* that do not require shikimic acid have been developed^31^,^32^, but manufacturing costs must be reduced before these routes will be practical. The patent on *Tamiflu* will expire in 2016, resulting in the likely widespread production of the generic drug. Therefore, shortages and price increases of shikimic acid are thought to be imminent.

Scale-up of the present method using mushroom mycelia and blue light stimulation is expected to yield high purity shikimic acid, since less than 1% quinic acid and 5% 3-dehydroshikimic acid, and no 3-dehydroquinic acid or protocatechuic acid, were detected (Fig. 3)^24^. This is a distinct advantage over recombinant microbial biocatalysis^27^. Moreover, it is estimated that the illumination of 1 kg of oyster mushroom mycelia with blue light for 36 h would produce about 0.45 g of shikimic acid. Therefore, the annual production of shikimic acid per kg of mycleia would be (0.45 g/1.5 d × 365 d/year), or 110 g. This is 1.4–3.7 times the amount of shikimic acid isolated from 1 kg of the current source, the plant *Illicium* *verum* (1 kg/year × 3–8/100 (the isolation efficiency) = 30–80 g)^33^,^34^. Critically, there is no obvious limitation on scale-up for the growth and blue light irradiation of mushroom mycelia or on the number of harvests per year, whereas the cultivation of *I. verum* is limited by land availability and is restricted to one harvest per year.

Finally, the finding that blue light stimulation affects the concentration of primary metabolites in oyster mushroom mycelia also suggests that light stimulation is an important environmental cue in filamentous fungi and other microorganisms for the regulation of primary and secondary metabolic pathways, particularly the aromatic amino acid pathway. Therefore, the present results are expected to motivate further research into photoresponses in microorganisms and their potential application in industry^24^.

## Methods

### Cultivation and Irradiation of Oyster Mushroom Mycelia

Modified MA medium consisted of 10 g of malt extract, 10 g of D(+)-glucose, 4 g of yeast extract and 25 g of agar in 1 liter of distilled water. GPY medium consisted of 50 g of D(+)-glucose, 2.5 g of polypeptone, 1.0 g of KH_2_PO_4_, 0.5 g of MgSO_4_ 7H_2_O, 0.5 g of CaCl_2_ 2H_2_O, 10 mg of FeCl_2_ 6H_2_O, 7.2 mg of MnCl_2_ 4H_2_O, 4.0 mg of ZnCl_2_, 1.0 mg of CuSO_4_ 5H_2_O, 2.5 g of yeast extract and 25 g of agar in 1 liter of distilled water. The initial pH was adjusted to 5.5. Media were autoclaved at 121°C for 20 min before use. Oyster mushroom dikaryotic strain (KH-3; Chikumakasei Co., Ltd., Nagano, Japan) was first incubated at 20°C in the dark on MA medium in a Pyrex Petri dish (diameter, 9 cm). The fungal thread was grown concentrically to form a mycelial colony about 70 mm in diameter. A colony 6 mm in diameter was excised from the periphery of the mycelial colony and inoculated onto GPY medium in the center of a Petri dish. Experiments on the effects of light stimulation on the production of primary metabolites, and for the analysis of gene expression and protein expression, were conducted using colonies about 65 mm in diameter grown on GPY agar media at 20°C in the dark.

An ELUX-1096 LED lighting unit for plant cultivation research (CCS, Inc., Kyoto, Japan) was used to culture the mycelia^35^, and the temperature was kept constant at 20°C. Light intensity was set using photon flux density (PFD) measurements taken with an LI-190 quantum sensor (LI-COR Biosciences, Inc., Lincoln, NE).

Mycelial colonies about 65 mm in diameter grown on GPY agar media in Pyrex Petri dishes at 20°C in the dark were wrapped with a film of polymethylpentene (JCCU, Tokyo, Japan) and further kept in the dark at 15°C, or irradiated immediately at 15°C for 0.5–72 h using blue LEDs at a PFD of 150 μmol m^−2^ s^−1^. Primary metabolites in the mycelia, and gene expression and protein expression, were then analyzed. The temperature shift from 20°C to 15°C in the dark did not result in the accumulation of shikimic acid, as seen in Figs. 2a and 2c. Wrapping the Petri dishes with the film improved the reproducibility of the primary metabolite analysis data, probably due to increased air supply to the mycelia. Irradiated and non-irradiated colonies at corresponding time points were immediately frozen in liquid nitrogen and stored at −80°C until use.

### Analysis of Primary Metabolites

Frozen tissue (approximately 50 mg) was plunged into 500 μL of methanol containing 50 μM internal standards (H3304-1002; Human Metabolome Technologies (HMT), Inc., Tsuruoka, Japan) at 0°C in order to inactivate the enzymes^36^. Tissue was homogenized three times at 1,500 rpm for 120 s using a tissue homogenizer (BMS-M10N21; Bio Medical Science Co., Ltd., Tokyo, Japan), then 500 μL of chloroform and 200 μL of Mill-Q water were added. Homogenate solution was centrifuged at 2,300 × *g* at 4°C for 5 min. Subsequently, 400 μL of the upper aqueous layer was centrifugally filtered through a Millipore (Bedford, MA) 5-kDa cutoff filter at 9,100 × *g* at 4°C for 120 min to remove proteins. The filtrate was then centrifugally concentrated and re-suspended in 50 μL of Milli-Q water for CE-MS analysis. Metabolome measurements were carried out through a facility service at HMT, Inc.

Capillary electrophoresis time-of-flight mass spectrometry (CE-TOF-MS) was carried out using an Agilent CE Capillary Electrophoresis System equipped with an Agilent 6210 Time of Flight mass spectrometer, an Agilent 1100 isocratic HPLC pump, an Agilent G1603A CE-MS adapter kit, and an Agilent G1607A CE-ESI-MS sprayer kit (Agilent Technologies, Waldbronn, Germany). Systems were controlled using Agilent G2201AA ChemStation software version B.03.01 for CE (Agilent Technologies). Metabolites were analyzed by using a fused silica capillary (50 μm *i.d.* × 80 cm total length), with commercial electrophoresis buffer (Solution ID: H3301-1001 for cation analysis and I3302-1023 for anion analysis; HMT, Inc.) as the electrolyte. Samples were injected at a pressure of 50 mbar for 10 s for cation analysis (approximately 10 nL sample) and 25 s for anion analysis (approximately 25 nL sample). The spectrometer was scanned from *m/z* 50 to 1,000. Other conditions were as described in references^37^,^38^,^39^.

Peaks were extracted using the automatic integration software MasterHands (Keio University, Tsuruoka, Japan) to obtain peak information, including *m/z*, migration time (MT) CE-TOF-MS measurement, and peak area^40^. Signal peaks corresponding to isotopomers, adduct ions and other product ions of known metabolites were excluded, and the remaining peaks were annotated with putative metabolites from the HMT metabolite database based on their MTs and *m/z* values determined by TOF-MS. The tolerance range for peak annotation was configured at ± 0.5 min for MT and ± 10 ppm for *m/z*. In addition, peak areas were normalized against those of the internal standards, and the resultant relative area values were further normalized by sample amount.

Hierarchical cluster analysis (HCA) and principal component analysis (PCA) were performed using HMT proprietary software (PeakStat and SampleStat, respectively). Detected metabolites were plotted on metabolic pathway maps using VANTED (Visualization and Analysis of Networks containing Experimental Data) software^41^. The HCA results are represented as a heatmap with standardized scores, which are averaged metabolite levels divided by standard deviation. Red indicates higher values, green indicates lower values, and white indicates no change for the non-irradiated mycelia compared to the blue-light irradiated mycelia. The color bar is automatically computed using maximum and minimum standardized values.

### Extraction and Analysis of Shikimic Acid using HPLC

Approximately 50 mg of each mushroom mycelial sample was immediately frozen in liquid nitrogen and stored at −80°C until metabolite extraction. The sample was completely homogenized using a freeze-crush apparatus (SK-100, Tokken, Inc., Chiba, Japan). After adding 0.5 mL of methanol, 0.5 mL of chloroform and 0.2 mL of Milli-Q water to the crushed sample, the solution was vortexed and homogenized using a homogenizer (Polytron PT10-35GT; Central Scientific Commerce, Inc., Tokyo, Japan) and a generator shaft (Polytron PT-DA 05/2EC-B078; Central Scientific Commerce, Inc.). Homogenate was centrifuged at 2,300 × *g* for 5 min at 4°C, then the supernatant was centrifugally filtered through a 5-kDa cut-off filter (Millipore) at 9,100 × *g* for 4 h at 4°C in order to remove proteins. The filtrate was concentrated using nitrogen gas and then dissolved in 0.5 mL of Milli-Q water. Shikimic acid was analyzed using a GL Sciences Inertsil HILIC column (5020-07736; GL Sciences Inc., Tokyo, Japan) and a Shimadzu LC workstation (Shimadzu Corp., Kyoto, Japan), consisting of a LC-10ADvp pump, a SIL-20A autosampler, a CTO-10ASvp column oven, and a SPD-10Avp UV detector (monitoring UV wavelength: 210 nm).

### Total RNA Isolation

Total RNAs were extracted from frozen colonies with an RNeasy Plant Mini Kit (Qiagen, Tokyo, Japan) in accordance with the manufacturer's instructions. Contaminating genomic DNA was removed using an RNase-Free DNase Set (Qiagen). The quantity and quality of total RNAs were evaluated with a Nanodrop ND-1000 spectrophotometer (Themo Fisher Scientific Inc., Waltham, MA) and an Agilent Bioanalyzer 2100 (Agilent Technologies, Palo Alto, CA), as recommended by the manufacturer.

**Analysis of Gene Expression using a Custom Microarray** (Accession Number of the National Center for Biotechnology Information (NCBI AN), GSE58522):

### cRNA Amplification and Labeling

Total RNA was amplified and labelled with Cyanine 3 (Cy3) using an Agilent Low Input Quick Amp Labeling Kit, one-color (Agilent Technologies), in accordance with the manufacturer's instructions. Briefly, 100 ng of total RNA was reverse transcribed into double-stranded cDNA using a poly dT-T7 promoter primer. Primer, template RNA and quality-control transcripts of known concentration and quality were first denatured at 65°C for 10 min, then incubated for 2 h at 40°C with 5 × first-strand Buffer, 0.1 MDTT, 10 mM dNTP mix and AffinityScript RNase Block Mix. The AffinityScript enzyme was inactivated at 70°C for 15 min. cDNA products were then used as templates for in vitro transcription to generate fluorescent cRNA. cDNA products were mixed with a transcription master mix in the presence of T7 RNA polymerase and Cy3 labeled-CTP, and were incubated at 40°C for 2 h. Labeled cRNA was purified using a Qiagen RNeasy mini spin column, then eluted with 30 μL of nuclease-free water. After amplification and labeling, cRNA quantity and cyanine incorporation were determined using a Nanodrop ND-100 spectrophotometer and an Agilent Bioanlyzer 2100.

### Sample Hybridization

For hybridization, 1.65 μg of Cy3-labeled cRNA was fragmented and hybridized at 65°C for 17 h to an Agilent GE8x15K custom microarray (NCBI AN, GPL18806). After washing, the microarrays were scanned using an Agilent DNA microarray scanner.

### Custom Microarray

DNA sequences for 12,330 predicted transcript models were obtained from the Joint Genome Institute (Pleurotus Ostreatus PC15 v2.0, http://genome.jgi-psf.org/PleosPC15_2/PleosPC15_2.home.html). Microarray probes were designed using Agilent eArray (https://earray.chem.agilent.com/earray/). A total of 12,244 probes were designed for 12,244 target transcripts (one probe per transcript).

### Analysis of Microarray Data (NCBI AN, GSM1412941 – 1412948)

The intensity values of each scanned feature were quantified using Agilent Feature Extraction software ver. 10.7.3.1, which performs background subtractions. We only used features that were flagged as no errors (present flags) and excluded features that were not positive, not significant, not uniform, not above background, saturated or population outliers (marginal and absent flags). Data were normalized using Agilent GeneSpring GX ver. 11.0.2 (per chip: normalization to 75 percentile shift; per gene: normalization to median of all samples). There were a total of 12,244 probes on an Agilent GE8x15K custom microarray without control probes. Altered transcripts were quantified using the comparative method. In this study, we used greater than or equal to 2-fold changes in signal intensity to identify significant differences of gene expression. Differentially expressed genes were also subjected to K-means analysis using GeneSpring ver. 11.0.2 software. HCA shown in Supplementary Fig. S3 was performed using TIGR MeV software v4.8.1 (distance metric: Euclidean, linkage rule: average; http://www.tm4.org/mev.html).

### Analysis of Gene Expression using a Real-Time PCR System

Forward and reverse primers for quantitative PCR analysis were designed using Primer3Plus Ver. 2.3.6 (Supplementary Table S3)^26^. First-strand cDNAs were synthesized from total RNAs with a High Capacity cDNA Reverse Transcription Kit (Applied Biosystems, Tokyo, Japan). Relative gene expression levels were determined in SYBR® Green Fast mode with a StepOne™ Real-time PCR System (Applied Biosystems). Both procedures were carried out in accordance with the manufacturer's instructions. All reactions were run in triplicate and 18S rRNA was used as an internal control.

### Preparation of Polyclonal Primary Antibodies

Polyclonal primary antibodies against PFK in the glycolysis pathway, G6PD in the pentose phosphate pathway and three isozymes for DAHPS in the shikimic acid pathway were prepared by Medical & Biological Laboratories Co., Ltd. (Nagoya, Japan). The production method was as follows. Synthesized peptides (Supplementary Table S4) corresponding to the five target proteins were conjugated with Kehole Limpet Hemocyanin (KLH). The KLH-conjugated peptides were mixed sufficiently with Freund's complete adjuvant to give a suspension. An immunogen of the same quality was injected intradermally into five individual rabbits (white female, New Zealand) five times weekly, together with the KLH-conjugated peptide and Freund's incomplete adjuvant. After eight weeks, blood was collected to produce the maximum amount of serum. The antibodies produced were used without further purification.

### Analysis of Protein Expression

Proteins were extracted from mushroom mycelia using an EzRIPA Lysis Kit (WSE-7420; ATTO Corp., Tokyo, Japan), in accordance with the manufacturer's instructions. Total protein concentration was determined using a Pierce 660 nm protein assay kit (Thermo Scientific, Rockford, IL). Protein solution was then mixed with an equal amount of EzApply (AE-1430; ATTO Corp.) pretreatment reagent for SDS-PAGE; target proteins were analyzed using 30 μg of total proteins. SDS-PAGE was performed using a PAGERUN (AE-6531P; ATTO Corp.) electrophoresis apparatus and e-PAGEL (E-T520L; ATTO Corp.), together with two types of molecular weight marker: EzStandard PrestainBlue (AE-1450; ATTO Corp.) and MagicMark XP Western Protein Standard (LC5603; Life Technologies, Tokyo, Japan). Separated proteins were Western blotted using a blotting apparatus (AE-6677P; ATTO Corp.), a power unit (AE-8130; ATTO Corp.) and a blotting reagent kit (AE-1460; ATTO Corp.) on a PVDF membrane (WSE-4051; ATTO Corp.). The membrane was blocked using blocking reagent (AE-1477; ATTO Corp.), then polyclonal primary antibodies diluted at 1:1,000–10,000 were reacted with the target proteins. Subsequently, target proteins were detected using secondary antibody (goat anti-rabbit IgG-HRP SC-2004; Santa Cruz Biotechnology, Inc., Santa Cruz, CA), Western blotting detection reagents (NCI3106; Thermo Scientific), and a luminescent image analyzer (ImageQuant LAS 4000 mini; GE Healthcare, Tokyo, Japan).

## Author Contributions

M.K. conceived of the project, designed the experiments, analyzed the data and wrote the manuscript. N.K. performed and analyzed gene and protein expression analysis. R.M. performed HPLC analysis.

## Supplementary Material

Supplementary Information Supplementary Information
