# Supplementary material for: Regulation of Primary Metabolic Pathways in Oyster Mushroom Mycelia Induced by Blue Light Stimulation: Accumulation of Shikimic Acid
Source: Sci Rep. 2015 Feb 27;5:8630. doi: 10.1038/srep08630 (PMC4342562; doi:10.1038/srep08630)
Supplement: Supplementary Information [file srep08630-s1.pdf]

Regulation of Primary Metabolic Pathways in Oyster Mushroom Mycelia Induced by Blue Light Stimulation: Accumulation of Shikimic Acid

Masanobu Kojima*, Ninako Kimura & Ryuhei Miura

Department of Bioscience and Biotechnology, Shinshu University, Kamiina, Nagano 399-4598, Japan.

*Correspondence and requests for materials should be addressed to M.K. (mkojima@shinshu-u.ac.jp).

Legends:

Supplementary Fig. S1. HPLC analysis of shikimic acid in oyster mushroom mycelia cultivated in the dark (before irradiation) and under blue light stimulation for 24 h.

Supplementary Fig. S2. Comprehensive analysis of gene expression in oyster mushroom mycelia after blue light stimulation using a custom microarray: PFD, 150 μmol m^{-2} s^{-1}; stimulation time, 0, 0.5, 1, 3, 6, 12, 24 and 36 h. **a**–**h**. Altered transcripts were quantified using the comparative method. Genes with greater than or equal to 2-fold changes in signal intensity were used to identify significant differences in gene expression. Differentially expressed genes were also subjected to K-means analysis and were classified into eight clusters (I–VIII). Total number of genes (TNG) categorized in each cluster is shown in **a**–**h**.

Supplementary Fig. S3. HCA of the time course of gene expression related to aromatic amino acid biosynthesis, glycolysis/gluconeogenesis and the pentose phosphate pathway.

Supplementary Fig. S4. Glycolysis pathway. EC2.7.1.11 (PFK) is a rate-determining enzyme.

Supplementary Fig. S5. Pentose phosphate pathway. EC1.1.1.49 (G6PD) is a rate-determining enzyme.

Supplementary Fig. S6. Time course of the expression levels of the rate-determining enzymes as detected by antibody response. The expression levels of the enzymes were analyzed using ImageJ software; n = 3; error bar = SD.

Supplementary Table S1. HCA of the time course induction of 212 primary metabolites by blue light stimulation

Supplementary Table S2. Probe names of genes represented in the custom microarray, EC number and the KEGG Orthology (KO) ID of the corresponding protein

Supplementary Table S3. Forward and reverse primers synthesized for quantitative PCR analysis

Supplementary Table S4. Peptides synthesized for preparation of polyclonal primary antibodies against the target proteins

Before irradiation

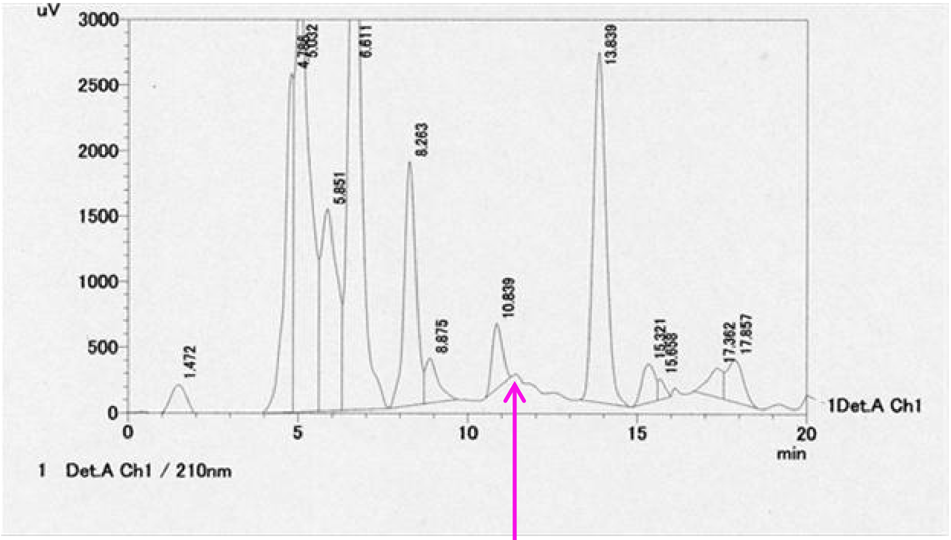

Analysis Conditions：
Column：Inertsil HILIC (GL Sciences Ltd.)
Particle Size: 5 μm
Length: Φ4.6 x 250 mm
Detection UV Wavelength: 210 nm
Mobile Phase: 1% H_3PO_4/MeCN = 10/90
Flow Rate: 0.6 ml/min
Oven Temp.: 30°C
Injection Amount: 10 μL
Retention Time of Shikimic Acid: 11.5 min

After blue light stimulation for 24 h

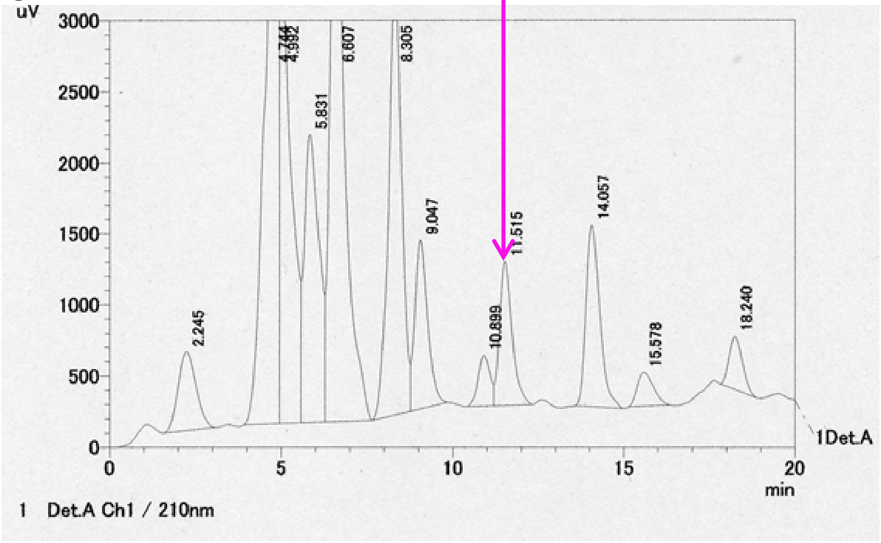

Supplementary Fig. S1

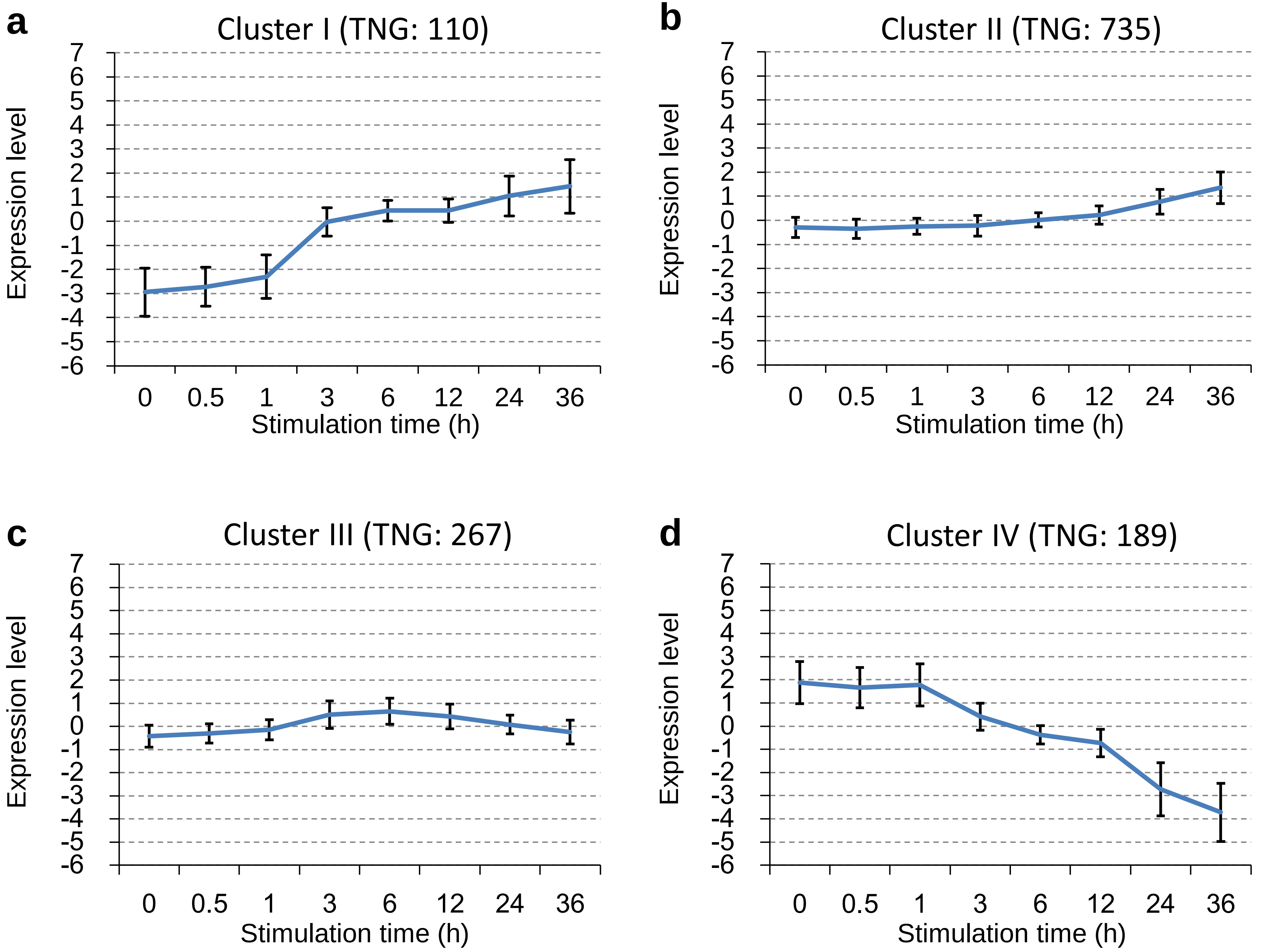

Supplementary Fig. S2a-d

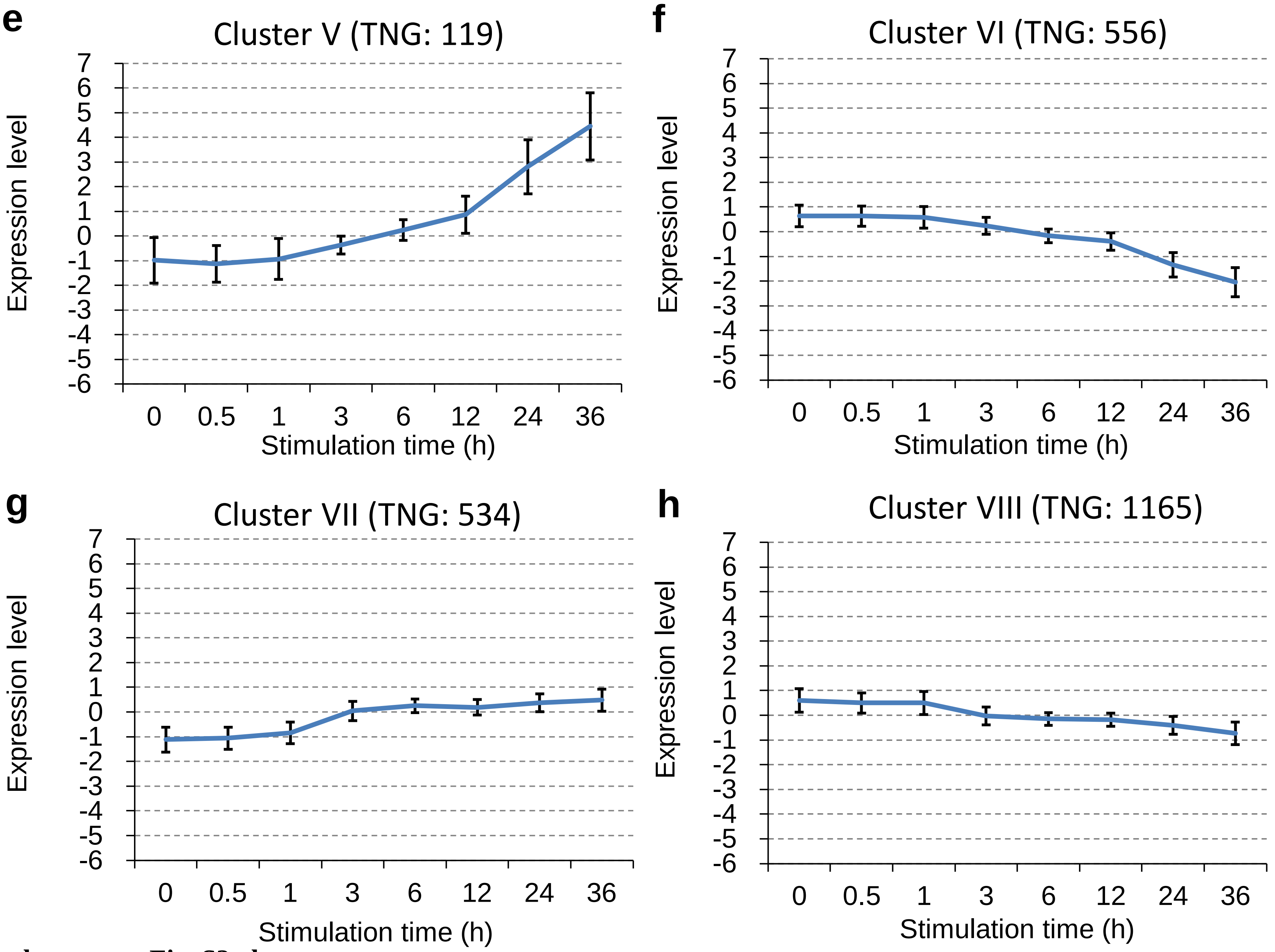

Supplementary Fig. S2e-h

HCA of the Time Course of Gene Expression Related to Phenylalanine, Tyrosine, Tryptophan Biosynthesis (Cf. KEGG map00400)

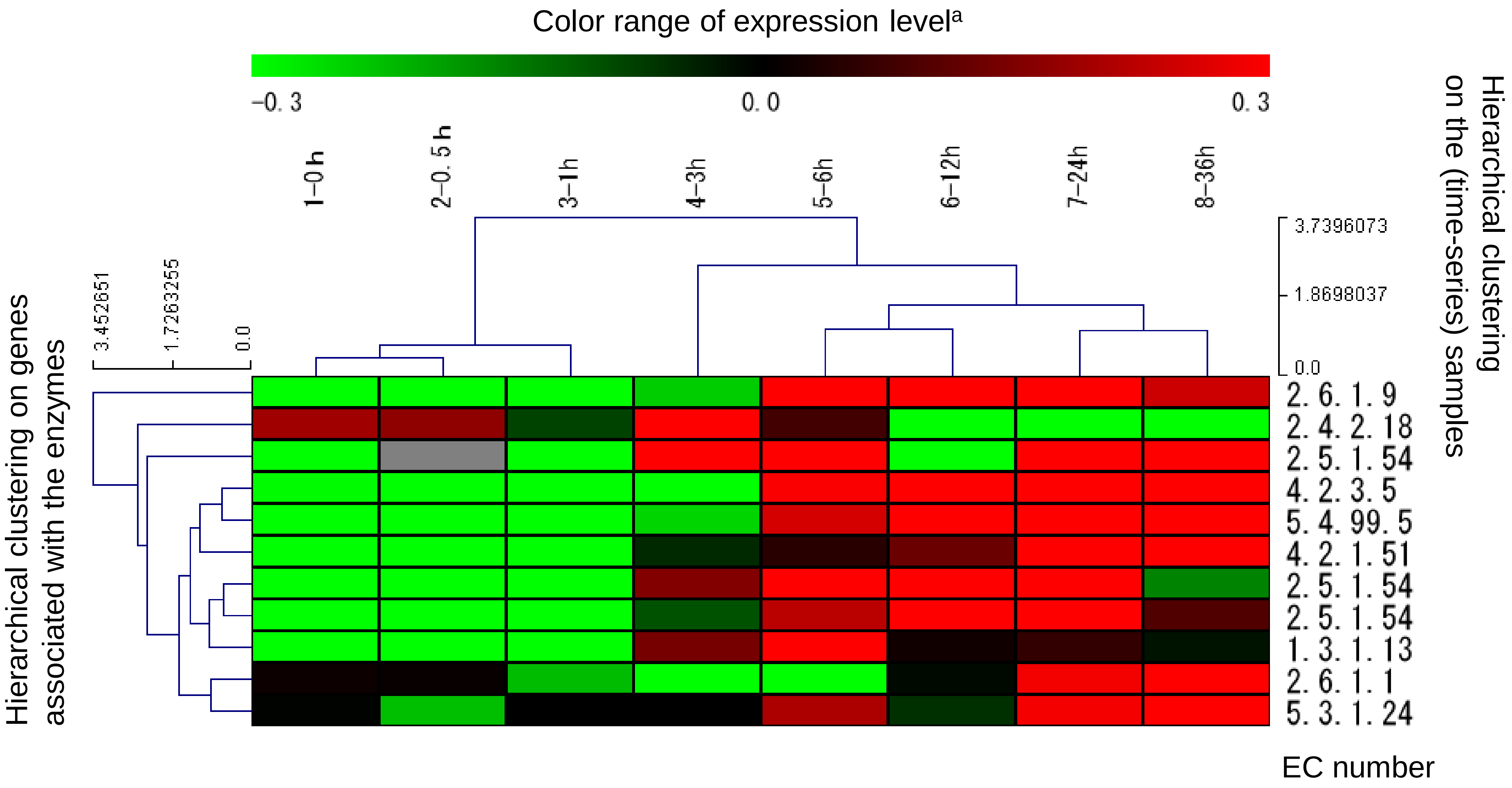

[a]Red and green cells indicate the ratio of each expression level above and below the median, respectively.

Supplementary Fig. S3a

HCA of the Time Course of Gene Expression Related to Glycolysis/Gluconeogenesis (Cf. KEGG map00010)

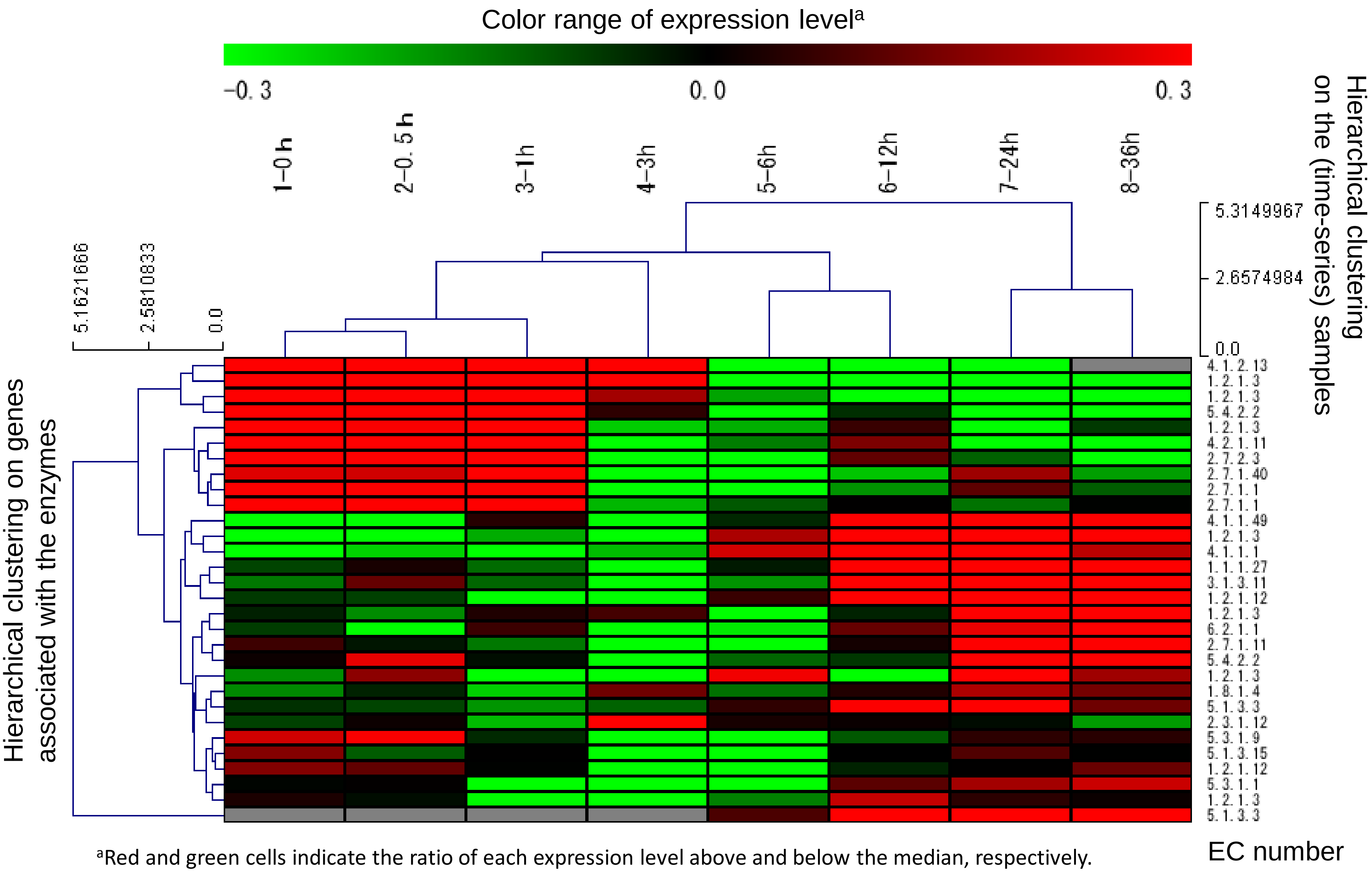

[a]Red and green cells indicate the ratio of each expression level above and below the median, respectively.

Supplementary Fig. S3b

HCA of the Time Course of Gene Expression Related to the Pentose Phosphate Pathway (Cf. KEGG map00030)

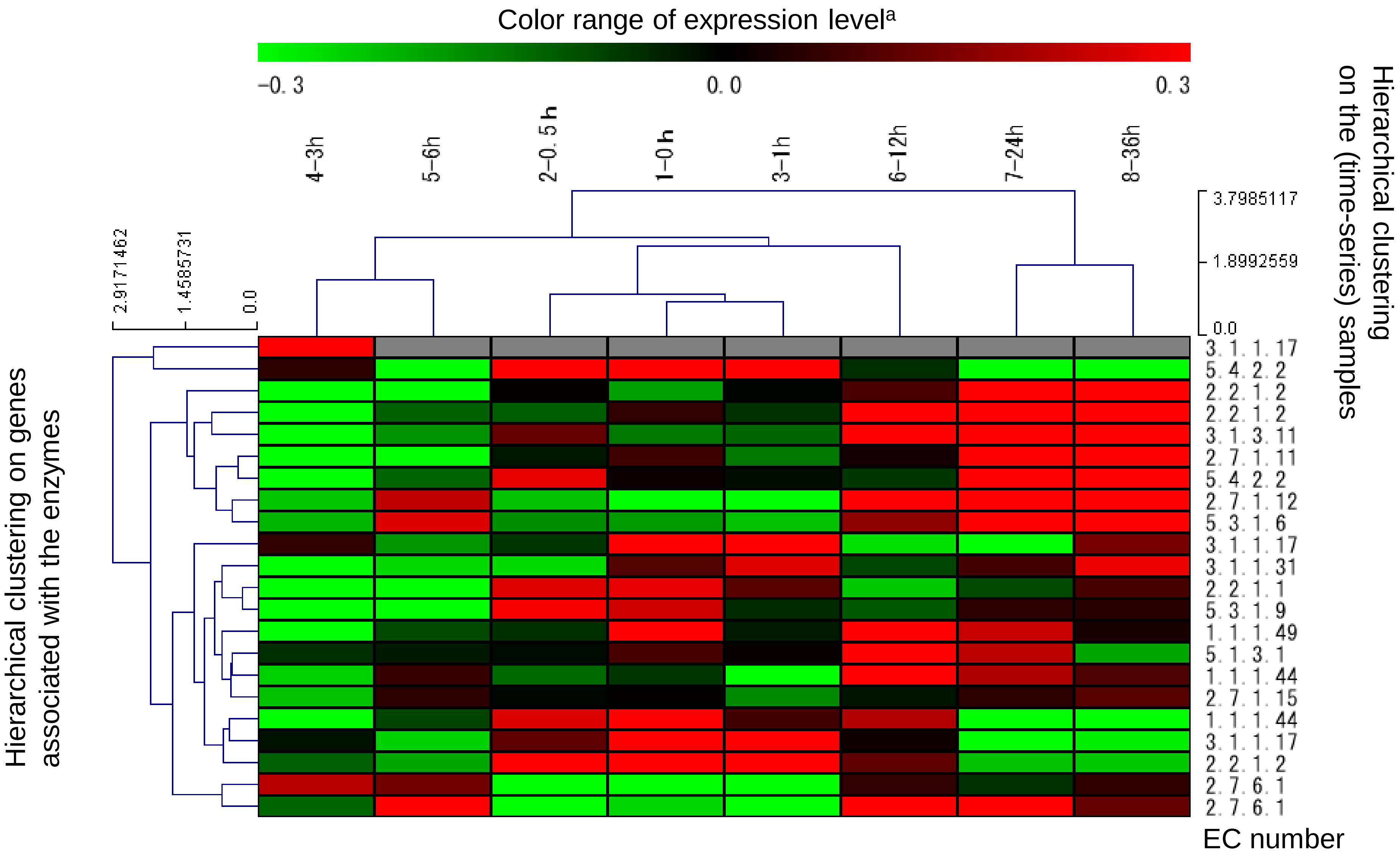

[a]Red and green cells indicate the ratio of each expression level above and below the median, respectively.

Supplementary Fig. S3c

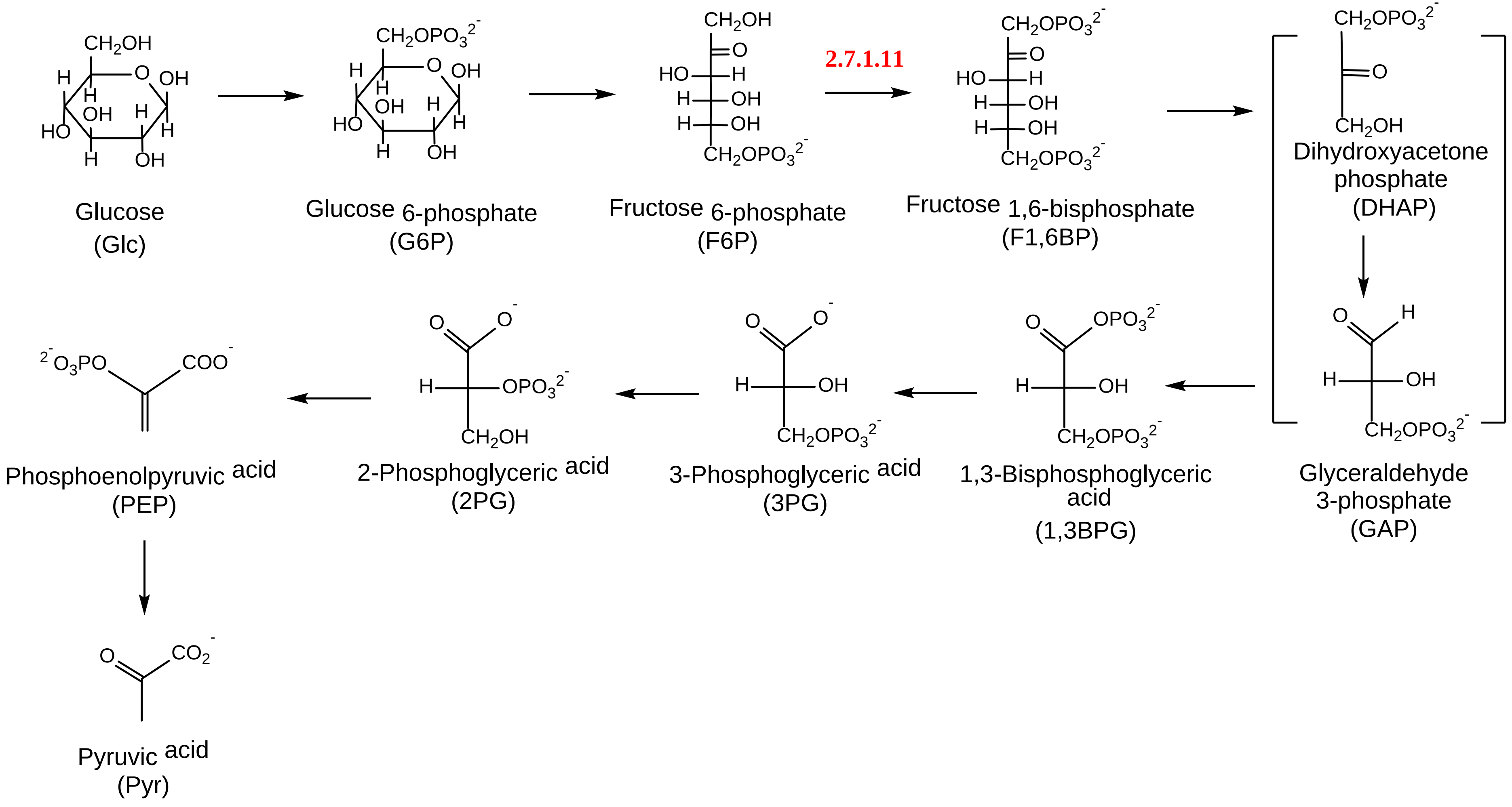

Supplementary Fig. S4

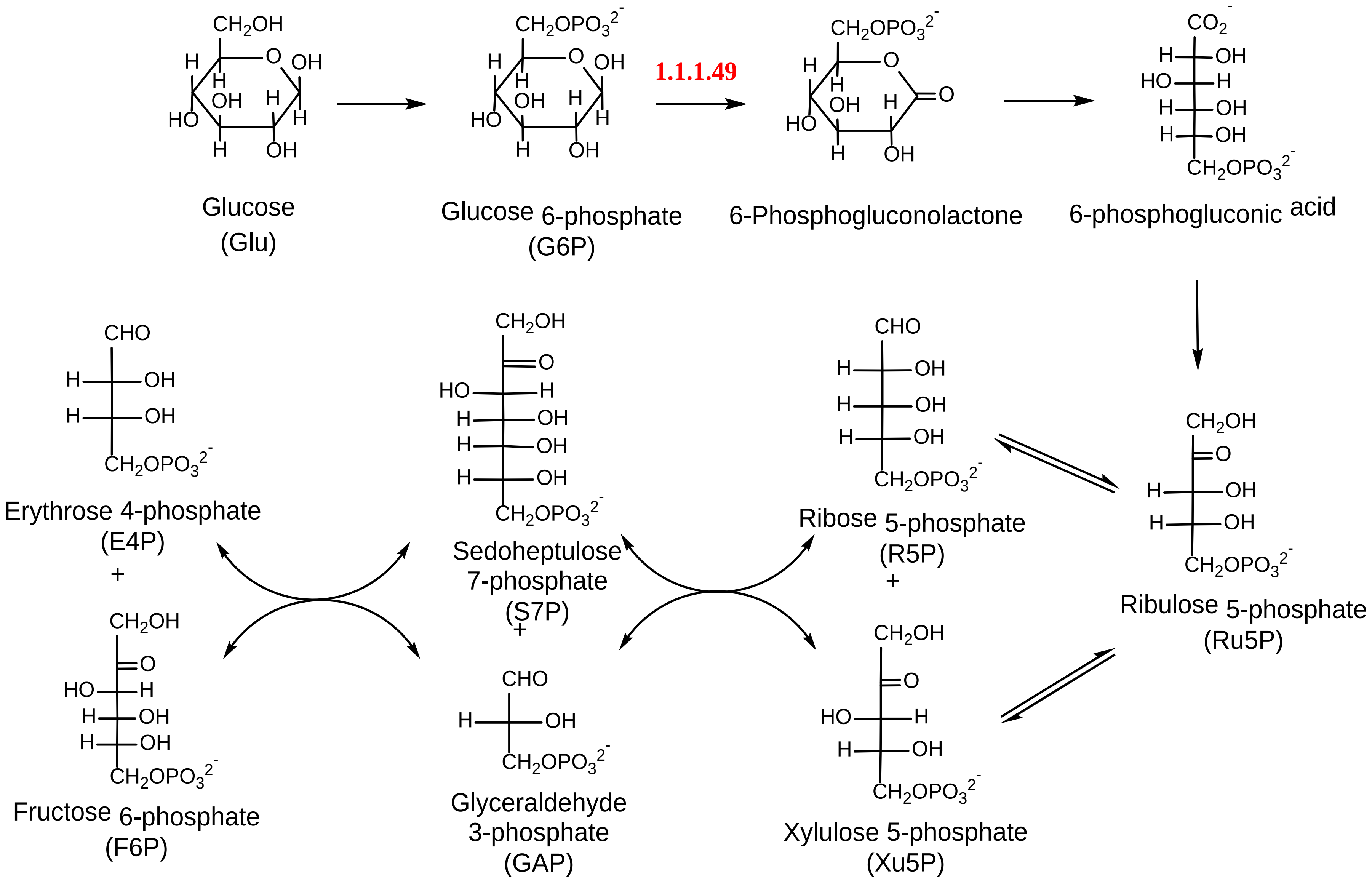

Supplementary Fig. S5

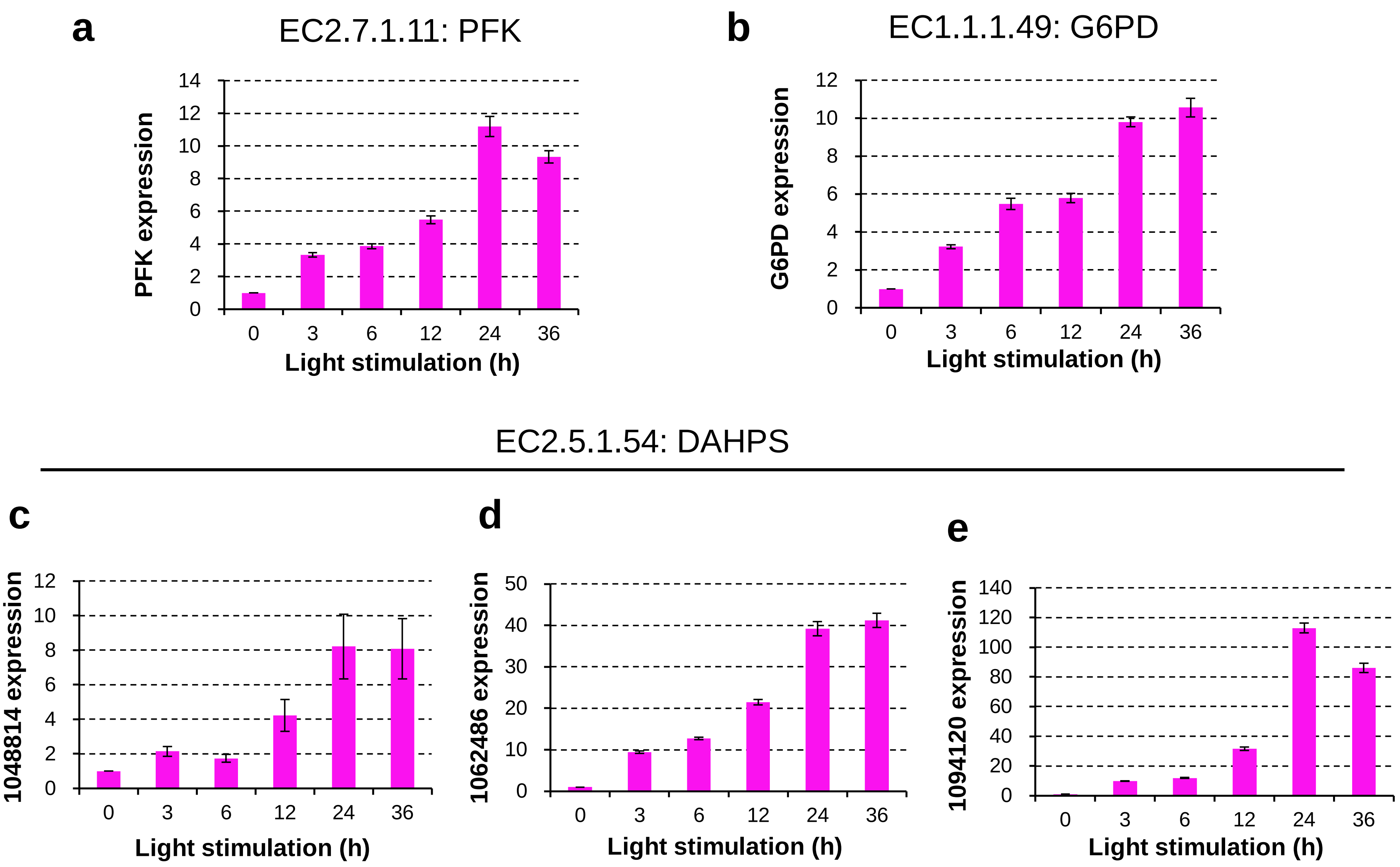

Supplementary Fig. S6

Supplementary Table S1. HCA of the Time Course Induction of 212 Primary Metabolites Induced by Blue Light Stimulation

NO.	Compound name	Blue-Light Stimulation Time vs Standardized Score					Dark
		0 h	6 h	12 h	24 h	36 h	36 h
1	γ-Glu-2-aminobutyric acid	2.04	-0.29	-0.36	-0.50	-0.47	-0.42
2	Phosphorylcholine	2.03	-0.33	-0.42	-0.57	-0.46	-0.26
3	XC0016 (Unknown)	2.03	-0.24	-0.58	-0.47	-0.36	-0.38
4	NADPH_divalent	2.02	-0.36	-0.39	-0.53	-0.56	-0.18
5	N-Acetylglutamic acid	2.02	-0.17	-0.52	-0.58	-0.36	-0.39
6	Citrulline	2.01	-0.15	-0.27	-0.58	-0.50	-0.51
7	2-Isopropylmalic acid	2.01	-0.39	-0.49	-0.55	-0.52	-0.06
8	2-Aminobutyric acid	1.99	-0.18	-0.30	-0.82	-0.33	-0.35
9	XA0033 (Unknown)	1.98	-0.29	-0.41	-0.72	-0.54	-0.03
10	3-Hydroxybutyric acid	1.97	0.00	-0.69	-0.60	-0.46	-0.23
11	N6-Acetyllysine	1.97	-0.36	-0.11	-0.63	-0.13	-0.75
12	S-Adenosylmethionine	1.97	-0.29	-0.44	-0.78	-0.49	0.03
13	N-Acetylornithine	1.97	-0.09	-0.24	-0.69	-0.73	-0.22
14	Fructose 1,6-diphosphate	1.96	0.01	-0.38	-0.71	-0.68	-0.21
15	Cystathionine	1.96	-0.30	-0.07	-0.89	-0.25	-0.46
16	Adenylosuccinic acid	1.94	-0.43	-0.54	-0.59	-0.60	0.22
17	Lys	1.93	-0.25	0.12	-0.34	-0.72	-0.74
18	myo-Inositol 2-phosphate	1.91	-0.98	-0.41	-0.07	-0.01	-0.45
19	Saccharopine	1.91	0.25	-0.39	-0.69	-0.75	-0.34
20	Hypotaurine	1.89	-0.17	-0.35	-1.07	0.05	-0.35
21	Hexanoic acid	1.89	-0.44	-0.44	-0.58	-0.78	0.34
22	N-Acetylglucosamine 6-phosphate	1.89	-0.19	-0.45	-0.83	-0.67	0.25
23	Argininosuccinic acid	1.89	-0.29	-0.58	-0.65	-0.70	0.33
24	Uric acid	1.86	-0.27	-0.63	-0.74	-0.60	0.38
25	Ornithine	1.86	-0.55	-0.33	0.16	-1.05	-0.09
26	5-Aminovaleric acid	1.82	-0.30	0.35	-0.61	-0.25	-1.01
27	Betaine	1.81	-0.41	-0.78	-0.51	-0.63	0.53
28	β-Ala	1.78	-0.40	-0.70	-0.80	-0.46	0.58
29	Ophthalmic acid	1.77	-0.05	-0.02	-0.67	0.14	-1.17
30	Choline	1.75	-0.22	-0.21	-1.27	0.31	-0.36
31	Glucose 1-phosphate	1.75	-0.21	-0.52	-0.74	-0.87	0.58
32	His	1.75	-0.10	0.53	-0.72	-0.52	-0.94
33	N-Acetylserine	1.71	0.32	-0.55	-0.68	-1.07	0.27
34	UMP	1.69	0.25	-0.29	-0.81	-1.12	0.29
35	Betaine aldehyde_+H2O	1.68	0.04	0.05	-0.90	0.26	-1.14
36	Glucose 6-phosphate	1.68	0.14	-0.28	-0.93	-1.02	0.41
37	m-Hydroxybenzoic acid	1.68	-0.67	-0.73	-0.80	-0.19	0.72
38	Butyrylcarnitine	1.67	0.25	-0.38	-1.36	-0.37	0.18
39	Arg	1.67	0.11	-0.27	0.09	-1.45	-0.16
40	ADP-glucoseGDP-fucose	1.65	-0.07	-0.26	-0.98	-0.94	0.59
41	3-Aminobutyric acid	1.65	0.28	0.48	-0.70	-0.79	-0.93
42	O-Phosphoserine	1.63	-0.24	-0.62	-0.69	-0.88	0.80
43	S-Adenosylhomocysteine	1.61	-0.90	-0.41	-0.07	0.72	-0.95
44	Threonic acid	1.60	-0.17	-0.78	-0.89	-0.58	0.82
45	Fructose 6-phosphate	1.60	0.17	-0.22	-0.99	-1.06	0.50
46	Sucrose 6'-phosphate	1.59	-0.09	-0.53	-0.81	-0.95	0.79
47	Uracil	1.58	-1.03	-1.13	0.04	0.19	0.35
48	3-Phosphoglyceric acid	1.58	0.69	-0.35	-1.00	-0.95	0.03
49	N,N-Dimethylhistidine	1.57	0.12	0.65	-0.86	-0.38	-1.11
50	Carboxymethyllysine	1.56	0.08	0.39	-0.40	-0.12	-1.49
51	3-Aminoisobutyric acid	1.56	0.16	-0.68	-1.09	-0.65	0.70
52	Homocitrulline	1.50	0.28	0.55	-0.19	-1.11	-1.04
53	XC0089 (Unknown)	1.49	-0.69	-0.61	-0.60	-0.66	1.07
54	Glycerol 3-phosphate	1.46	0.11	-0.85	-0.42	-1.13	0.82
55	CDP-choline	1.41	-0.43	-0.25	-0.72	-1.06	1.05
56	Pro	1.38	-0.09	0.24	-0.60	0.60	-1.52
57	Dihydroxyacetone phosphate	1.33	0.95	-0.11	-1.18	-0.95	-0.05
58	UDP-glucoseUDP-galactose	1.33	0.01	-0.44	-0.97	-0.99	1.06
59	Ergothioneine	1.31	-0.14	-0.53	-1.03	-0.77	1.16
60	myo-Inositol 1-phosphate<br>myo-Inositol 3-phosphate	1.29	0.18	-0.90	-0.30	-1.23	0.95
61	UDP-glucuronic acid	1.29	0.19	-0.23	-1.12	-1.06	0.93
62	Sedoheptulose 7-phosphate	1.26	0.12	-0.40	-0.87	-1.17	1.06
63	Uridine	1.26	-1.20	-1.17	0.71	0.26	0.15
64	N-Acetylneuraminic acid	1.24	-1.14	-0.86	0.78	-0.64	0.62
65	2-Phosphoglyceric acid	1.23	1.07	-0.09	-1.23	-0.90	-0.08
66	Homoserine	1.22	-0.92	-0.13	-0.07	1.10	-1.20
67	dATP	1.12	-0.64	-0.64	-0.64	-0.64	1.45
68	dTDP	1.12	-0.88	0.42	-0.88	-0.88	1.11
69	Histidinol	1.06	-0.48	-0.78	-0.71	-0.57	1.48
70	Erythrose 4-phosphate	1.05	0.09	-0.30	0.05	-1.75	0.86
71	Sebacic acid	1.04	-0.62	-0.79	-0.55	-0.58	1.51

72	GDP	1.03	0.44	0.25	-0.98	-1.48	0.74
73	UDP	0.99	0.81	-0.01	-1.22	-1.21	0.65
74	dTTP	0.98	-0.63	-0.63	-0.63	-0.63	1.56
75	Hydroxyproline	0.98	0.27	0.44	0.29	-0.06	-1.92
76	Cys	0.94	-0.20	1.46	-1.16	-0.34	-0.71
77	Adenine	0.93	0.20	-1.12	-1.12	1.25	-0.15
78	GMP	0.93	-1.41	-0.38	1.18	0.37	-0.69
79	10-Hydroxydecanoic acid	0.89	-0.34	-0.77	-0.57	-0.80	1.59
80	CoA_divalent	0.86	-0.28	1.53	-1.10	-0.74	-0.26
81	UTP	0.86	0.56	-0.28	-1.15	-1.12	1.14
82	Citric acid	0.85	0.05	-0.40	-1.11	-0.85	1.47
83	Ribulose 5-phosphate	0.78	0.88	-0.72	-0.96	-1.03	1.05
84	Glutathione (GSH)	0.75	-0.56	-0.52	-0.49	-0.86	1.69
85	GABA	0.73	-0.71	-1.25	-0.68	1.20	0.70
86	2-Aminoadipic acid	0.71	-0.32	1.62	-0.17	-1.03	-0.81
87	6-Phosphogluconic acid	0.71	0.07	0.08	-0.81	-1.41	1.36
88	N-Formylaspartic acid	0.71	-0.36	0.57	0.28	-1.88	0.67
89	Pantothenic acid	0.64	-0.61	-0.80	-0.39	-0.60	1.75
90	O-Succinylhomoserine	0.58	0.79	0.89	0.20	-0.95	-1.52
91	Val	0.56	-0.10	0.20	-0.54	1.42	-1.53
92	GTP	0.56	0.69	0.05	-1.07	-1.34	1.12
93	Creatine	0.44	0.14	-0.76	-0.91	-0.64	1.73
94	Ethanolamine	0.38	-0.96	-1.41	0.13	0.64	1.22
95	ATP	0.36	0.64	0.17	-1.06	-1.34	1.23
96	Malic acid	0.23	0.02	-0.39	-0.54	-1.12	1.80
97	N-Acetylaspartic acid	0.21	-0.50	-0.48	-0.58	1.95	-0.59
98	Cadaverine	0.19	0.89	1.01	-0.95	0.33	-1.47
99	FAD_divalent	0.18	-0.64	-0.73	-0.43	-0.31	1.93
100	Trp	0.18	-0.73	0.02	-0.60	-0.74	1.88
101	2-Methylserine	0.13	-0.39	-0.80	-0.71	-0.15	1.92
102	ADP	0.10	0.66	0.77	-0.61	-1.72	0.79
103	Glucuronic acid	0.07	-0.45	-0.07	-0.30	-1.10	1.86
104	N-Acetylasparagine	0.06	0.80	0.51	-0.82	-1.55	1.01
105	Phe	0.04	0.43	0.12	0.32	-1.92	1.01
106	Leu	0.04	-0.12	0.24	-0.10	1.54	-1.60
107	Cytidine	-0.02	0.36	0.74	1.26	-1.23	-1.10
108	Carnitine	-0.03	-0.22	-0.73	-0.76	-0.21	1.95
109	Asp	-0.03	0.22	0.02	-1.67	1.47	-0.01
110	N-Acetylglycine	-0.06	-0.48	-0.49	-0.44	2.01	-0.55
111	cGMP	-0.09	-0.60	-0.49	0.52	-1.07	1.72
112	dTDP-glucose	-0.10	0.59	0.63	-1.10	-1.24	1.22
113	Fumaric acid	-0.11	0.27	-0.28	-0.57	-1.12	1.80
114	4-Methyl-2-oxovaleric acid<br>3-Methyl-2-oxovaleric acid	-0.13	0.38	-0.56	0.32	1.49	-1.49
115	CTP	-0.14	1.37	0.26	-1.10	-1.13	0.74
116	UDP-N-acetylglucosamine	-0.21	0.79	0.48	-1.05	-1.24	1.21
117	XA0002 (Unknown)	-0.21	-1.93	0.39	0.47	0.52	0.76
118	CDP	-0.22	1.45	0.82	-0.94	-1.14	0.03
119	Trigonelline	-0.23	-0.52	-1.17	-0.38	0.64	1.66
120	Ribose 5-phosphate	-0.23	1.89	-0.04	-0.70	-0.95	0.04
121	N-Acetylmethionine	-0.24	0.03	-0.52	1.67	-1.32	0.38
122	Terephthalic acid	-0.25	-0.55	1.95	-0.01	-0.87	-0.27
123	NMN	-0.27	-0.52	-0.83	0.16	-0.46	1.93
124	Gln	-0.29	-0.51	-0.36	0.15	-0.91	1.92
125	Met	-0.32	-0.50	-0.22	-0.24	2.01	-0.72
126	Spermidine	-0.41	0.16	-0.17	-0.67	-0.82	1.91
127	cAMP	-0.42	-0.71	-0.71	1.06	-0.71	1.48
128	3-Hydroxy-3-methylglutaric acid	-0.43	-0.55	-0.22	0.00	1.97	-0.77
129	Tryptamine	-0.45	-0.62	-0.66	-0.08	-0.17	1.98
130	Oxypurinol	-0.45	-0.54	-0.56	-0.15	2.02	-0.32
131	5-Oxoproline	-0.49	-0.39	-1.35	1.37	0.95	-0.09
132	N-Acetylleucine	-0.50	-0.79	-0.38	0.87	1.60	-0.80
133	Pipecolic acid	-0.51	0.21	1.28	0.97	-0.65	-1.30
134	Trimethylamine	-0.52	-0.64	-0.70	0.73	1.72	-0.58
135	Trehalose 6-phosphate	-0.53	1.00	0.29	-1.46	1.15	-0.45
136	4-Guanidinobutyric acid	-0.54	-0.67	-0.71	0.38	-0.33	1.87
137	XC0040 (Unknown)	-0.56	-0.01	-0.02	-0.08	-1.16	1.83
138	3-Methylhistidine	-0.59	-0.07	0.27	0.53	-1.53	1.39
139	Isocitric acid	-0.60	-0.52	-0.08	-0.47	2.01	-0.33
140	CMP-N-acetylneuraminate	-0.60	-1.18	-0.40	0.69	1.61	-0.11
141	β-Ala-Lys	-0.61	-0.67	-0.77	1.24	-0.52	1.33
142	Phenylpyruvic acid	-0.62	-0.58	-0.30	-0.41	2.00	-0.10
143	cCMP2',3'-cCMP	-0.62	-0.62	-0.62	1.66	-0.62	0.84
144	XA0004 (Unknown)	-0.64	0.06	-0.65	0.99	-1.14	1.37
145	Glucosaminic acid	-0.65	-0.83	-0.80	0.20	1.76	0.31
146	Galacturonic acid	-0.67	-0.75	0.03	0.67	1.64	-0.92
147	3-Hydroxypropionic acid	-0.68	-0.78	0.44	1.83	-0.23	-0.58
148	2,4-Diaminobutyric acid	-0.68	0.96	-0.57	1.41	-1.14	0.03

149	2-Phenylethylamine	-0.69	-0.47	-0.45	1.99	-0.01	-0.37
150	Lactic acid	-0.71	-0.43	-0.37	-0.26	-0.24	2.01
151	SDMA	-0.72	-1.05	0.25	1.04	1.27	-0.78
152	Inosine	-0.75	-0.46	0.05	0.63	1.62	-1.09
153	AMP	-0.78	-0.66	0.53	1.81	-0.40	-0.50
154	Putrescine	-0.78	1.08	0.50	1.08	-0.85	-1.03
155	Ile	-0.79	-0.09	0.36	0.23	1.58	-1.30
156	Imidazole-4-acetic acid	-0.80	0.88	1.06	0.62	-1.39	-0.37
157	γ-Butyrobetaine	-0.80	-1.00	0.00	-0.33	1.76	0.37
158	4-Acetamidobutanoic acid	-0.83	0.36	-0.38	-0.02	-0.92	1.79
159	Formylanthranilic acid	-0.84	-0.47	-0.24	0.33	1.87	-0.65
160	trans-Glutaconic acid	-0.84	0.70	0.27	-0.70	-0.96	1.52
161	Adenosine	-0.86	-0.62	-0.32	1.45	1.07	-0.72
162	Shikimic acid	-0.87	-0.58	0.09	0.55	1.68	-0.88
163	Glutathione (GSSG)_divalent	-0.88	-0.77	-0.25	0.58	1.75	-0.43
164	ADMA	-0.89	-0.65	-0.09	1.39	1.07	-0.84
165	Digalacturonic acid	-0.89	0.13	-0.28	1.39	-1.20	0.86
166	Nω-Methylarginine	-0.89	-0.16	0.61	1.72	-0.50	-0.78
167	CMP	-0.91	1.45	1.08	-0.43	-0.48	-0.71
168	Cysteine glutathione disulfide	-0.94	0.00	0.21	0.29	1.62	-1.17
169	Ala-Ala	-0.95	-0.13	-0.25	1.88	-0.68	0.13
170	Gly	-0.95	-0.41	-0.02	0.29	1.82	-0.73
171	Methionine sulfoxide	-0.96	-0.43	-0.40	0.84	1.61	-0.66
172	Imidazolelactic acid	-0.98	0.31	0.95	1.32	-0.78	-0.82
173	Ser	-0.98	0.27	0.28	-0.71	-0.58	1.74
174	Isethionic acid	-1.02	-0.50	-0.14	0.38	-0.53	1.81
175	Gluconolactone	-1.02	-0.22	0.10	1.35	0.91	-1.12
176	Suberic acid	-1.02	-0.99	-0.21	1.66	0.35	0.21
177	3'-Dephospho CoA	-1.03	-0.66	0.00	1.72	0.51	-0.54
178	XC0017 (Unknown)	-1.04	-0.39	-0.14	0.91	1.50	-0.83
179	N5-Ethylglutamine	-1.04	-0.67	0.78	1.49	0.21	-0.77
180	Succinic acid	-1.05	-0.42	0.64	1.05	0.93	-1.16
181	2-Hydroxyvaleric acid	-1.05	1.18	0.67	0.84	-0.82	-0.82
182	N6,N6,N6-Trimethyllysine	-1.05	-0.84	-0.20	1.71	0.46	-0.08
183	Gluconic acid	-1.06	-0.21	0.17	1.41	0.79	-1.09
184	Guanine	-1.06	0.97	-0.21	1.40	-0.16	-0.95
185	Arg-Glu	-1.07	-0.45	-0.02	1.81	-0.54	0.27
186	Glycerophosphocholine	-1.11	-0.35	0.54	-0.62	1.71	-0.17
187	Allantoic acid	-1.12	-0.87	0.02	0.73	-0.29	1.54
188	Isovalerylcarnitine	-1.14	-0.06	0.39	1.41	0.53	-1.13
189	Isobutyrylcarnitine	-1.15	0.22	1.18	0.22	0.79	-1.26
190	N-Acetyllysine	-1.15	-0.22	0.20	1.83	-0.28	-0.38
191	O-Acetylcarnitine	-1.17	-0.88	-0.44	1.03	1.24	0.22
192	Tyr	-1.18	-0.94	-0.37	0.25	0.95	1.28
193	Sorbitol 6-phosphate	-1.19	-0.18	-0.26	0.47	-0.56	1.72
194	XC0065 (Unknown)	-1.20	-0.31	0.21	0.76	1.43	-0.89
195	Serotonin	-1.25	-0.32	0.09	1.75	0.19	-0.47
196	XC0126 (Unknown)	-1.28	-0.31	-0.02	1.08	1.26	-0.72
197	Guanosine	-1.29	0.08	0.16	1.50	0.48	-0.92
198	Glycerol	-1.35	0.30	0.27	0.39	1.37	-0.98
199	Trimethylamine N-oxide	-1.37	0.62	-0.98	1.28	0.15	0.31
200	N-Acetylglucosamine 1-phosphate	-1.40	-0.21	0.16	0.83	1.33	-0.71
201	Thr	-1.41	-0.12	0.54	0.15	1.49	-0.66
202	Asn	-1.42	-0.89	-0.21	0.64	0.93	0.94
203	Gly-Asp	-1.45	-0.31	-0.08	1.67	0.09	0.08
204	NAD+	-1.46	-0.32	-0.40	-0.05	1.31	0.93
205	Imidazole-4-methanol	-1.53	-0.45	-0.43	1.24	0.44	0.73
206	Phthalic acid	-1.58	0.35	1.41	0.26	-0.57	0.13
207	NADP+	-1.60	-0.38	0.57	0.88	0.99	-0.46
208	Glyceric acid	-1.60	-0.05	0.36	1.04	0.91	-0.65
209	N-Carbamylglutamic acid	-1.61	0.21	0.13	1.08	-0.67	0.86
210	cis-Aconitic acid	-1.68	-0.20	0.35	-0.35	1.19	0.69
211	Carnosine	-1.81	-0.19	0.16	1.18	0.13	0.52
212	Pyruvic acid	-1.87	0.88	0.11	0.20	0.81	-0.12

Supplementary Table S2. Probe Names of Genes Represented in the Custom Microarray, EC Number and the KEGG Orthology (KO) ID of the Corresponding Protein

Phenylalanine, Tyrosine and Tryptophan Biosynthesis Pathway (KEGG Map00400)

EC number	Probe Name	KEGG ORTHOLOGY (KO) ID
1.3.1.13	CUST_1678_PI426540746	K00211
2.4.2.18	CUST_9044_PI426540746	K00766
2.5.1.54	CUST_8613_PI426540746<br>CUST_10556_PI426540746<br>CUST_3158_PI426540746	K01626
2.6.1.1	CUST_741_PI426540746	K14454
2.6.1.9	CUST_11589_PI426540746	K00817
4.2.1.51	CUST_3391_PI426540746	K04518
4.2.3.5	CUST_2990_PI426540746	K01736
5.3.1.24	CUST_6467_PI426540746	K13501
5.4.99.5	CUST_8552_PI426540746	K01850

Glycolysis/Guconeogenesis Pathway
(KEGG Map00010)

EC number	Probe Name	KEGG ORTHOLOGY (KO) ID
1.1.1.27	CUST_12058_PI426540746	K00016
1.2.1.12	CUST_10594_PI426540746	K00134
	CUST_10568_PI426540746	
1.2.1.3	CUST_10900_PI426540746	K00128
	CUST_10901_PI426540746	
	CUST_401_PI426540746	
	CUST_2225_PI426540746	
	CUST_11032_PI426540746	
	CUST_10890_PI426540746	
	CUST_5033_PI426540746	
1.8.1.4	CUST_11109_PI426540746	K00382
2.3.1.12	CUST_3370_PI426540746	K00627
2.7.1.1	CUST_4719_PI426540746	K00844
	CUST_475_PI426540746	
2.7.1.11	CUST_9760_PI426540746	K00850
2.7.1.40	CUST_3597_PI426540746	K00873
2.7.2.3	CUST_1231_PI426540746	K00927
3.1.3.11	CUST_832_PI426540746	K03841
4.1.1.1	CUST_8648_PI426540746	K01568
4.1.1.49	CUST_6433_PI426540746	K01610
4.1.2.13	CUST_8389_PI426540746	K01623
4.2.1.11	CUST_4128_PI426540746	K01689
5.1.3.15	CUST_3480_PI426540746	K01792
5.1.3.3	CUST_5430_PI426540746	K01785
	CUST_11314_PI426540746	
5.3.1.1	CUST_11091_PI426540746	K01803
5.3.1.9	CUST_6014_PI426540746	K01810
5.4.2.2	CUST_1244_PI426540746	K01835
	CUST_7339_PI426540746	
6.2.1.1	CUST_110_PI426540746	K01895

Pentose Phosphate Pathway
(KEGG map00030)

EC number	Probe Name	KEGG ORTHOLOGY (KO) ID
1.1.1.44	CUST_2935_PI426540746	K00033
	CUST_11042_PI426540746	
1.1.1.49	CUST_4747_PI426540746	K00036
2.2.1.1	CUST_1169_PI426540746	K00615
2.2.1.2	CUST_8317_PI426540746	K00616
	CUST_11449_PI426540746	
	CUST_1056_PI426540746	
2.7.1.11	CUST_9760_PI426540746	K00850
2.7.1.12	CUST_6006_PI426540746	K00851
2.7.1.15	CUST_6355_PI426540746	K00852
2.7.6.1	CUST_3501_PI426540746	K00948
	CUST_3110_PI426540746	
3.1.1.17	CUST_11803_PI426540746	K01053
	CUST_11809_PI426540746	
	CUST_7102_PI426540746	
3.1.1.31	CUST_3275_PI426540746	K01057
3.1.3.11	CUST_832_PI426540746	K03841
5.1.3.1	CUST_3595_PI426540746	K01783
5.3.1.6	CUST_10980_PI426540746	K01807
5.3.1.9	CUST_6014_PI426540746	K01810
5.4.2.2	CUST_1244_PI426540746	K01835
	CUST_7339_PI426540746	

Supplementary Table S3. Forward and Reverse Primers Synthesized for Quantitative PCR Analysis

EC / Protein ID	Forward Primer	Reverse Primer
EC2.7.1.11 / 174437	5'-TCA GCA ATA ACG TCC CGA TG-3'	5'-TTG CTT GAT CGC ATC ACA GG-3'
EC1.1.1.49 / 1088946	5'-ACG TTC AAG GAA CCG TTT GG-3'	5'-ACC TGC AGC AAA TGG TTC TG-3'
EC2.5.1.54 / 1048814	5'-ACA CAT CCA AGA TGC GTT CG-3'	5'-AAT TGC CAT GGG AGC AAT CG-3'
EC2.5.1.54 / 1062486	5'-TTC TGA GAT CGA ACG CTT GC-3'	5'-TTT CAT GAG TGC AGG CAT CG-3'
EC2.5.1.54 / 1094120	5'-ACG CCA AAC TCC TCA AAC AG-3'	5'-TTG TTC GGG GCT TTT CGA AG-3'

Supplementary Table S4. Peptides Synthesized for Preparation of Polyclonal Primary Antibodies against the Target Proteins

Target protein	EC / Protein ID	AMW[a] / kDa	Synthesized peptide
phosphofructokinase (PFK)	EC2.7.1.11 / 174437	94.4	CDNPGTTNYGEGTGQDTE
glucose-6-phosphate dehydrogenase (G6PD)	EC1.1.1.49 / 1088946	56.5	SDSGTIPSMETSHDEC
3-deoxy-D-arabinoheptulosonate 7-phosphate (DAHP) synthase	EC2.5.1.54 / 1048814	43.9	MGKPLVPDFSDDEKVC
3-deoxy-D-arabinoheptulosonate 7-phosphate (DAHP) synthase	EC2.5.1.54 / 1062486	54.1	CEQNNLLYSELGGRERKP
3-deoxy-D-arabinoheptulosonate 7-phosphate (DAHP) synthase	EC2.5.1.54 / 1094120	40.7	CRQGVRGRRELLKKQARP

[a]Average molecular weight
